# Assessing and Exploiting Functional Diversity in Germplasm Pools to Enhance Abiotic Stress Adaptation and Yield in Cereals and Food Legumes

**DOI:** 10.3389/fpls.2017.01461

**Published:** 2017-08-29

**Authors:** Sangam L. Dwivedi, Armin Scheben, David Edwards, Charles Spillane, Rodomiro Ortiz

**Affiliations:** ^1^Independent Researcher Hyderabad, India; ^2^School of Biological Sciences, Institute of Agriculture, University of Western Australia, Perth WA, Australia; ^3^Plant and AgriBiosciences Research Centre, Ryan Institute, National University of Ireland Galway Galway, Ireland; ^4^Department of Plant Breeding, Swedish University of Agricultural Sciences Alnarp, Sweden

**Keywords:** crop improvement, epigenetic variation, florigen pathways, functional diversity, genome editing, genomic estimated breeding value, haplotypes, TILLING

## Abstract

There is a need to accelerate crop improvement by introducing alleles conferring host plant resistance, abiotic stress adaptation, and high yield potential. Elite cultivars, landraces and wild relatives harbor useful genetic variation that needs to be more easily utilized in plant breeding. We review genome-wide approaches for assessing and identifying alleles associated with desirable agronomic traits in diverse germplasm pools of cereals and legumes. Major quantitative trait loci and single nucleotide polymorphisms (SNPs) associated with desirable agronomic traits have been deployed to enhance crop productivity and resilience. These include alleles associated with variation conferring enhanced photoperiod and flowering traits. Genetic variants in the florigen pathway can provide both environmental flexibility and improved yields. SNPs associated with length of growing season and tolerance to abiotic stresses (precipitation, high temperature) are valuable resources for accelerating breeding for drought-prone environments. Both genomic selection and genome editing can also harness allelic diversity and increase productivity by improving multiple traits, including phenology, plant architecture, yield potential and adaptation to abiotic stresses. Discovering rare alleles and useful haplotypes also provides opportunities to enhance abiotic stress adaptation, while epigenetic variation has potential to enhance abiotic stress adaptation and productivity in crops. By reviewing current knowledge on specific traits and their genetic basis, we highlight recent developments in the understanding of crop functional diversity and identify potential candidate genes for future use. The storage and integration of genetic, genomic and phenotypic information will play an important role in ensuring broad and rapid application of novel genetic discoveries by the plant breeding community. Exploiting alleles for yield-related traits would allow improvement of selection efficiency and overall genetic gain of multigenic traits. An integrated approach involving multiple stakeholders specializing in management and utilization of genetic resources, crop breeding, molecular biology and genomics, agronomy, stress tolerance, and reproductive/seed biology will help to address the global challenge of ensuring food security in the face of growing resource demands and climate change induced stresses.

## Assessing Crop Functional Diversity

Producing sufficient food for the growing population is a major challenge, with climate change emerging as an additional threat to the food security and livelihood of millions of people (Abberton et al., 2016). Achieving significant yield gains in staple crops is essential because rising demand requires a twofold increase in crop production by 2050 (Tilman et al., 2011). The increasing frequency of droughts and heat stress is impacting crop productivity (Deryng et al., 2014; Lesk et al., 2016), and the increased frequency and severity of flooding events may cause yield loss in regions such as Asia, where prolonged flooding of rice fields already substantially reduces yields (Mackill et al., 2012). To meet the challenges of increasing demand in a changing climate, there is a need to more rapidly generate new and improved crop cultivars.

Cereals and grain legumes constitute the major components of the human diet and of livestock feed. Grain legumes also enrich soil with nitrogen and improve soil texture for other crops (Graham and Vance, 2003). The discovery of semi-dwarfing genes fuelled the stark increase in yields (known as the ‘Green Revolution’) in rice and wheat production globally (Trethowan et al., 2007). However, the reliance on a narrow range of elite cultivars has likely led to some negative effects on agroecosystems productivity (Dwivedi et al., 2017), though this assumption remains controversial and empirical research provides contradictory evidence (Fu, 2015). More recent evidence also suggests that productivity of major food crops is either stagnating or not increasing at the rate needed to ensure food security (Ortiz, 2015). Accelerated progress in plant breeding is required to better harness crop genetic resources and produce higher-yielding, climate-resilient cultivars.

As the methods to assess functional diversity in crops have become more sophisticated during the last 100 years, our understanding of the mechanisms underlying this diversity has grown. Functional diversity refers to a component of biodiversity related to what organisms do in communities and ecosystems (Petchey and Gaston, 2006). The decreasing cost of high-throughput DNA sequencing has facilitated the recent rise of genome-wide methods such as genotyping by sequencing (Scheben et al., 2017a) for assessing functional diversity of crops using single nucleotide polymorphisms (SNPs) (Kilian and Graner, 2012; Huang and Han, 2014). Common targets of breeding are yield-related traits such as abiotic stress tolerance, pest resistance and flowering time. The potential yield gains are substantial, considering that abiotic stress can reduce average yields of major crops by 50% (Bray et al., 2000) and pests can cause 26–40% yield losses (Oerke, 2006). Assessing and using functional diversity in pathways controlling flowering time is also important for yield, particularly as control of crop development can enhance adaptation to the predicted impact of climate change. The genomics era has led to a rapid increase in sequence data capturing the genetic diversity underlying heritable target traits in elite cultivars, landraces and crop wild relatives. However, while there were already over 100 plant genomes available in 2015 (Michael and VanBuren, 2015), over half of which were crops, the functions of the vast majority of plant genes remain unknown (Rhee and Mutwil, 2014).

Powerful and high-throughput forward and reverse genetic techniques are required to help elucidate these unknown gene functions to assist targeted breeding. Genetic mapping approaches also play an important role in associating genomic regions with phenotypic traits. Vast improvements in our understanding of the functional knowledge of crop genomes is an important prerequisite for targeted genome editing based approaches to access novel diversity for breeding programs (Scheben et al., 2017b), which often remain limited by the natural diversity found in germplasm resources. Both understanding and shaping of crop functional diversity using genomic technologies will be necessary to ensure continuing yield increases to keep pace with growing global food demand. In this review article, we focus on the latest developments in assessing and exploiting functional diversity associated with abiotic stress adaptation, phenology, plant architecture, and yield attributing traits in cereals and food legumes germplasm pools using genomics-led methods for crop genetic enhancement. We focus on three questions: (1) How do we characterize functional diversity? (2) What are the key breeding targets? (3) How can we apply knowledge of functional diversity to improve crop traits using genomic prediction and genome editing?

## Approaches for Uncovering Functional Diversity

Analysis of DNA variation regulating phenotypes (traits) in crops can facilitate the identification of causal genes associated with desired agronomic traits. Advances in genome sequencing have dramatically reduced costs of measuring DNA variation, facilitating the identification of candidate genes for complex traits. To date, many crop genomes are sequenced, yielding millions of SNPs, while resequencing of diverse germplasm (including wild species) across crop genepools further provides a wealth of genomic information (in some instances related with discrete phenotypes). Single nucleotide polymporphisms (SNPs) are most abundant genetic markers that are amenable to automation and cost-effective for use and integration with crop breeding research. In particular, SNPs which are robustly associated with desirable agronomic phenotypes can provide a better understanding of gene function while also providing markers that can be used for more-efficient plant breeding schemes (Huq et al., 2016).

### Genome-Wide SNP Polymorphism

#### Legumes

Soybean (*Glycine max*) has been extensively investigated for SNP variation using diverse genepools (**Table 1**). Valliyodan et al. (2016) reported over 10 million high quality SNPs and 0.75 m InDels, mostly (82.6%) in intergenic regions. Wild soybeans had 15% more SNPs than landraces and elite lines. Soybean cultivars also showed high SNP polymorphism (dos Santos et al., 2016). SNP-based arrays in soybean include the SoySNP1.5K (Shen et al., 2005), SoySNP6K (Akond et al., 2013), SoySNP50K (Song et al., 2013), and 180 K AXIOM^®^ SoyaSNP (Lee et al., 2015) arrays. The SoySNP355K array, which covers the whole genome, is also available (Wang J. et al., 2016).

**Table 1:** Genome wide SNPs discovered in chickpea, common bean, cowpea, groundnut, pea, pigeonpea, and soybean.

Genome-wide SNPs, InDels and structural variants (SVs)	Germplasm source	Reference
**Chickpea (*Cicer arietinum*)**		
2,058,566 SNPs and 292,588 InDels	35 accessions representing 16 mapping populations	Thudi et al., 2016
CicArVarDB containing 1,965 803 SNPs and InDels	90 accessions	Doddamani et al., 2015
82,489 SNPs	93 wild and cultivated accessions	Bajaj et al., 2015
**Common bean (*Phaseolus vulgaris*)**		
6286 DArT Seq high density SNPs	188 accessions, including landraces and cultivars from Andean and Mesoamerican gene pools	Valdisser et al., 2017
44,875 SNPs, 3633 InDels	18 cultivated and wild accessions	Ariani et al., 2016
768-SNP Illumina GoldenGate assay	6 common bean and 2 tepary bean accessions	Gujaria-Verma et al., 2016
BARCBean6K_3 BeadChip containing 6000 SNPs	365 dry bean and 134 snap bean accessions	Song Q. et al., 2015
**Cowpea (*Vigna unguiculata*)**		
1,048 SNPs	768 accessions	Xiong et al., 2016
**Groundnut (*Arachis hypogaea*)**		
Affymetrix 60K SNP array ^±^	20 cultivated accessions	Clevenger et al., 2017
**Pea (*Pisum sativum*)**		
131,850 SNPs	4 accessions	Boutet et al., 2016
GenoPea 13.2K SNP Array	12 RIL populations	Tayeh et al., 2015
**Pigeonpea (*Cajanus cajan*)**		
4,686,422 SNPs and 779,254 InDels	20 accessions belonging to primary and secondary genepools	Kumar et al., 2016
**Soybean (*Glycine max*)**		
5,835,185 SNPs and 1,329,844 InDels	28 Brazilian cultivars	dos Santos et al., 2016
9,790,744 SNPs, 876,799 InDels	302 wild, landraces, and improved accessions	Zhou et al., 2015
Axiom^®^ SoyaSNP array containing 180 961 SNPs	47 accessions	Lee et al., 2015
10 million SNPs, including 35% not previously reported	106 accessions representing wild, landraces, and elite lines	Valliyodan et al., 2016
3,871,469 SNPs	10 cultivated and six wild accessions	Chung et al., 2014
5,102,244 SNPs and 707,969 insertion/deletions	55 accessions	Li et al., 2013
SoySNP50K array	6 cultivated and 2 wild accessions	Song et al., 2013
SoySNP6K BeadChip array containing 5,376 SNPs	92 RILs involving soybean cultivars ‘Maryland 96-5722’ and ‘Spencer’	Akond et al., 2013
205,614 SNPs	17 wild and 14 cultivated accessions	Lam et al., 2010
SoySNP1.5K chip array GoldenGate assay	Selected from 2,435 random SNPs evenly covering the genome from the Soybean SNP database	Shen et al., 2005

*^±^highly flexible for Arachis, with applications for genotyping tetraploid populations, interspecific populations, and intraspecific diploid populations*.

Clevenger et al. (2017) re-sequenced 20 diverse groundnut (*Arachis hypogaea*) accessions to identify SNP variations and constructed a large-scale genotyping array, which contains 58,233 putative SNPs, including those from groundnut ancestors *A. duranensis* (21,547 SNPs) and *A. ipaensis* (22,933 SNPs). The array is designed to be highly flexible for *Arachis*, with applications for genotyping *A. hypogaea* populations, interspecific populations, and intraspecific diploid populations. A unique feature of this array is its set of 1,193 SNPs indicative of tetrasomic recombination (i.e., tetrasomic inheritance) events. Thus, this newly developed SNP array will be very useful for further genetic and breeding applications in *Arachis*.

The RCBean6K_3BeadChip array containing 6,000 SNPs is widely used in beans (Song Q. et al., 2015). A gene-based SNP array in tepary bean (*Phaseolus acutifolius*) provided greater insight of this species’ population structure and its relationship with common bean (*Phaseolus vulgaris*), facilitating the introgression of agriculturally important traits (Gujaria-Verma et al., 2016). Development of high throughput genotyping arrays, GenoPea 13.2KSNP in pea (*Pisum sativum*) (Tayeh et al., 2015) and Axiom^®^*Cicer*50.6SNP array in chickpea (*Cicer arietinum*) (Roorkiwal et al., 2016), are expected to accelerate genetic research. The chickpea database repository CicArVarDB contains 1.9 million SNPs and InDels anchored on eight pseudomolecules, allowing to select for variation associated with quantitative trait loci (QTL) (Doddamani et al., 2015).

The International Cowpea Consortium and Illumina have developed a new SNP genotyping array for cowpea (*Vigna unguiculata*). This 60,000-marker iSelect array provides a 40-fold increase in marker density compared to an older, 1,536-marker GoldenGate Illumina panel (Close, 2015). Pigeonpea (*Cajanus cajan*) has lagged behind in array technology, though abundant SNPs have been identified (Kumar et al., 2016).

#### Cereals

Both maize (*Zea mays*) and rice (*Oryza sativa*) have been extensively studied for SNP variation using diverse germplasm (**Table 2**). A publicly available high-density SNP array (609,442 SNPs and 6,759 InDels) optimized for European and American temperate maize, the Affymetrix^®^ Axiom^®^ Maize Genotyping Array, was recently developed (Unterseer et al., 2014). MaizeSNP3072 array containing 3,072 SNPs is more efficient than MaizeSNP50 array in fingerprinting Chinese cultivars (Tian et al., 2015). A maize 55 K SNP array with improved genome coverage was developed on an Affymetrix^®^ Axiom^®^ platform with 55,229 SNPs evenly distributed across the genome, which contains 451 markers associated with 368 known genes including those for drought tolerance and kernel oil biosynthesis, 4067 markers not assigned to any chromosome or position in the current reference genome, 734 markers differentiating heterotic groups, and 132 markers tagged for important transgenic events. This array improves MaizeSNP50 (Ganal et al., 2011), and is a powerful tool for germplasm evaluation, marker-assisted breeding, QTL mapping and association studies for both tropical and temperate maize (Xu C. et al., 2017).

**Table 2:** Genome wide SNPs discovered in barley, maize, oat, pearl millet, rice, sorghum, and wheat.

Genome-wide SNPs, InDels and structural variants (SVs)	Germplasm source	Reference
**Barley (*Hordeum vulgare*)**		
1,688,807 SNPs; 143,872 InDels	267 georeferenced landraces and wild accessions	Russell et al., 2016
544,318 SNPs	433 wild and domesticated accessions	Pankin et al., unpublished
ISelect 9K chip consisting of 7,864 SNPs	a diverse panel of 804 contemporary barley cultivars having a spring or winter growth habit	Comadran et al., 2012
**Maize (*Zea mays*)**		
3,252,194 SNPs, 213,181 InDels, 39,631 SVs	A four-row waxy landrace accession	Liu H. et al., 2016
383,145 SNPs	Targeted sequencing of 29 Mb genomic regions with 4,648 genes linked with biomass in 21 inbred lines	Muraya et al., 2015
616,201 SNPs and InDels	30 temperate maize lines	Unterseer et al., 2014
6,385,011 SNPs	15 inbred lines	Xu et al., 2014
687,257 SNPs	2815 inbred lines from USDA genebank	Romay et al., 2013
MaizeSNP50 array	274 lines, including B73, Mo 17, NAM parents, and inbreds	Ganal et al., 2011
1,272,134 SNPs and 30,178 InDels	6 elite inbred lines	Lai et al., 2010
**Pearl millet (*Pennisetum glaucum*)**		
83,875 SNPs	500 accessions	Hu et al., 2015
**Rice (*Oryza sativa* L.)**		
976,791 SNPs and 46,640 InDels	RGD-7S and Taifeng B	Fu et al., 2016
Rice SNP50 (OsSNPnks) array	192 diverse accessions	Singh et al., 2015
Rice SNP50 (Illumina Infinium platform) array	801 accessions	Chen et al., 2014
RiceSNP6K	500 landraces	Yu et al., 2014
6,496,456 SNPs	40 cultivated and 10 wild accessions	Xu et al., 2012
**Sorghum (*Sorghum bicolor*)**		
∼265,000 SNPs	971 worldwide accessions	Morris et al., 2013
4,946,038 SNPs	44 accessions	Mace et al., 2013
1,957,018 SNPs, 99,948 InDels	3 accessions (sweet and grain sorghum)	Zheng et al., 2011
**Bread wheat (*Triticum aestivum*)**		
>4 million inter-varietal SNPs across chromosome 7	16 Australian cultivars	Lai et al., 2015
wheatSNP 90K array	726 accessions	Wang S. et al., 2014
9,000 gene-associated SNPs	2,994 accessions including landraces and modern cultivars	Cavanagh et al., 2013

Rice SNP50 array contains 51,478 evenly distributed markers (Chen et al., 2014). This array incorporates 50,051 SNPs from 18,980 single copy genes (3,710 conserved between wheat and rice, 14,959 unique to rice, 194 agronomically important cloned rice genes) and 117 multicopy rice genes, mapped on 12 rice chromosomes. The utility of this assay was demonstrated for genetic diversity and phylogenetic research, using a panel of diverse genepools, and in breeding (Singh et al., 2015).

A high-density array in wheat (*Triticum aestivum*) contains about 90,000 gene-associated SNPs from populations of diverse geographical origins. This array consists of 46,977 SNPs that were mapped using eight segregating populations (Wang S. et al., 2014).

Whole-genome resequencing (16–45× genome coverage) of 44 accessions of the diverse origins, end-use and taxonomic groups unravels 8 million high-quality SNPs and 1.9 million InDels in sorghum (*Sorghum bicolor*) (Mace et al., 2013), while resequencing three sorghum inbred lines uncovered 1 million SNPs, 0.099 million InDels, 0.106 million presence/absence variations, and 0.017 million copy number variations (Zheng et al., 2011). Sequencing of 500 pearl millet (*Pennisetum glaucum*) accessions identified 83,875 SNPs (Hu et al., 2015), while targeted resequencing of 433 diverse accessions generated a genome-wide panel of 544,318 high quality SNP in barley (*Hordeum vulgare*) (Pankin et al., unpublished).

Clearly, technological innovations in genomics have already led to discovery of abundant polymorphic SNPs in most cereal and legume crops, thus facilitating trait discovery and introgression. As the pace of technological advances and cost-reductions in next-generation sequencing technologies is extremely rapid, it is possible that SNP-array based platforms may be superseded by or become largely integrated with high-throughput sequencing approaches to genotyping (Pérez-Enciso et al., 2015).

### QTL and Candidate Genes for Complex Traits

Genome-wide association studies (GWAS) carried out on diversity panels can provide higher mapping resolution than linkage mapping based on biparental crosses, thus allowing better detection of candidate causal genes (Ingvarsson and Street, 2011; Huang and Han, 2014). GWAS success depends, however, on data quality, population size, and the degree of linkage disequilibrium (LD) (Flint-Garcia et al., 2005; Mackay and Powell, 2007). Mutation, population structure, epistasis, and population perturbations such as migration, inbreeding, and selection all affects LD (Jannink and Walsh, 2002). LD decay varies between species, among different populations within species, and among different loci within a given genome (Tenaillon et al., 2001; Gupta et al., 2005; Caldwell et al., 2006).

Multigenic complex traits such as plant architecture, yield and related traits, and stress adaptation are typically affected by many genes and are also influenced by genotype × environment interactions. GWAS has been successful for detecting natural variation underlying some complex traits which has enabled researchers to identify several associated SNPs, some of which were co-located with previously reported QTL or candidate genes.

#### Phenology and Pod/Seed Traits in Legumes

**Table 3** lists selected candidate genes for crop phenology in soybean and common bean or those associated with pod or seed characteristics in chickpea and cowpea. Zhang J. et al. (2015) reported new loci and refined genomic regions of known loci associated with crop duration (i.e., number of days from sowing to harvesting) and plant height in soybean. Candidate genes homologous to flowering genes in *Arabidopsis thaliana* were located near the peak SNP associated with flowering in soybean (Zhang J. et al., 2015). An allelic variant of the CesA-type cellulose synthase gene, *Ca_Kabuli_CesA3*, was found to regulate pod and seed numbers plant^-1^ in chickpea (Kujur et al., 2015). *Phvul.001G221100* was associated with days to flower and maturity in common bean (Kamfwa et al., 2015). SNPs were also identified which are associated with pod length in cowpea (Xu P. et al., 2017).

**Table 3:** SNPs and germplasm-based genome-wide association studies (GWAS) for phenology and yield in chickpea, common bean, cowpea and soybean.

Association mapping panel and SNPs	Summary of marker-trait association and candidate genes identified	Reference
**Chickpea (*Cicer arietinum*)**		
211 accessions; 44,844 SNPs	22 major loci associated with pods/seeds plant^-1^ and 100-seed weight; an allelic variants of CesA-type cellulose synthase gene, *Ca_Kabuli_CesA3*, regulated high pods/seeds plant^-1^, contributing 47% phenotypic variation	Kujur et al., 2015
**Common bean (*Phaseolus vulgaris*)**		
237 accessions; 5,398 SNPs	A candidate gene *Phvul.001G221100* on *P. vulgaris* (*Pv*) chromosome 01 associated with days to flower and maturity; significant SNPs for seed yield mapped on Pv03 and Pv09 colocalized with previously identified QTL for yield	Kamfwa et al., 2015
**Cowpea (*Vigna unguiculata*)**		
299 accessions; 50,000 SNPs	72 SNPs associated with pod length	Xu P. et al., 2017
**Soybean (*Glycine max*)**		
309 accessions; 50,000 SNPs	27, 6, 18, and 27 loci, respectively, associated with flowering, maturity, flowering to maturity duration, and plant height; *Dt1* strongly associated with maturity and plant height; a pectin lyase-like gene near the major for plant height locus	Zhang J. et al., 2015
139 accessions; 47,000 SNPs	1-8 loci associated with maturity, plant height, and seed weight, with most co-populated with priori known QTL affecting these traits	Sonah et al., 2014
168 landraces; 1,536 SNPs	51 SNPs associated with chlorophyll and chlorophyll fluorescence parameters, of which 14 co-associated with two or more traits and 8 with previously reported yield and yield components	Hao et al., 2012

#### Plant Architecture and Edible Yield in Cereals

Domestication and subsequent artificial selection by humans has dramatically changed plant architecture, phenology and components of grain yield in many cereals, largely to address agronomic needs and to adapt the crops to various stress-prone environments. Candidate genes and SNPs associated with crop phenology, plant architecture, and yield-attributing traits are known in cereals (**Table 4**). Several unique candidate gene regions related to plant growth and development and grain yield have been identified in maize (Farfan et al., 2015; Li X. et al., 2016). Bouchet et al. (2017) found 34 and 6 QTL for individual or combinatorial trait combinations in maize, respectively. They identified a QTL cluster in a 5 Mb region around *Tb1* associated with tiller number and ear row number. The latter was positively correlated with flowering (days to anthesis for male and female flowering and anthesis to silking interval measured in days) and negatively correlated to grain yield. *Kn1* and *ZmNIP1* have been identified as candidate genes for tillering, along with *ZCN8* for leaf number and *Rubisco Activase 1* for kernel weight. A more upright leaf in maize has been shown to be influenced by variation in *liguleless* genes (Tian et al., 2011).

**Table 4:** SNPs and germplasm-based genome-wide association studies for plant architecture traits in barley, maize, rice, sorghum, and wheat.

Association mapping panel and SNPs	Summary of marker-trait association and candidate genes identified	Reference
**Barley (*Hordeum vulgare*)**		
1420 nested association mapping panel; 7864 SNPs	Eight major QTL accounted for 64% variance associated to flowering, with strongest QTL effect corresponded to *Ppd-H1*	Maurer et al., 2015
224 accessions; 957 SNPs	171 significant marker-trait associations for agronomic traits delineated into 107 QTL (57 novel and 50 congruent QTL), populated with priori mapped QTL	Pasam et al., 2012
**Maize (*Zea mays*)**		
336 accessions; 50K SNP	34 QTL for individual and six for trait combinations, with only five pleiotropic; a cluster of QTL around *Tb1* associated with tiller and ear row numbers; candidate genes for tillering, leaf number and kernel weight	Bouchet et al., 2017
258 inbreds; 224,152 SNPs	41 SNPs associated with plant and ear height, of which 29 located in 19 unique candidate gene regions related to plant growth and development	Li X. et al., 2016
346 inbreds; 60,000 SNPs	10 quantitative trait variants associated with grain yield, plant and ear height, and flowering with some colocalizing to previously reported QTL	Farfan et al., 2015
NAM panel of 5000 RILs by crossing 25 diverse lines to a reference line; 1.6 m SNPs	Key genes with small effects (little epistasis, environmental interaction or pleiotropy) controlled leaf angle, leaf length and width; variations at the *liguleless* genes contribute to more upright leaves	Tian et al., 2011
**Rice (*Oryza sativa*)**		
225 accessions; 83,374 SNPs	56 SNPs associated with panicle architecture traits: 17, spikelets panicle^-1^; 10, primary branches; 11, secondary branches; 7, primary branch length; 11, secondary branch length	Rebolledo et al., 2016
242 accession; 700,000 SNPs	10 candidate genes regulate plant architecture, half of which overlap with QTL associated with panicle architecture traits	Crowell et al., 2016
315 accessions; 44,100 SNPs	7, 5, 10, 8, and 6 genomic regions associated with panicle architecture traits including grain characteristics	Ya-fang et al., 2015
529 accessions; 4,358,600 SNPs	141 associated loci for 15 agronomic traits; of which 25 mapped within known gene, i.e., *SD1*	Yang et al., 2014
950 cultivars; 4,109,366 SNPs	32 SNP loci associated with flowering and grain related traits; identified candidate genes for 18 associated loci	Huang et al., 2012
413 accessions; 44,100 SNPs	A dozen of common variants influencing numerous complex traits	Zhao et al., 2011
517 landraces; 3.6 million SNPs	The identified loci contributed ∼36% of the phenotypic variance, on average; six loci closely associated with previously identified genes	Huang et al., 2010
**Sorghum (*Sorghum bicolor*)**		
390 accessions; 268,830 SNPs	SNPs loci for grain yield, grain number, and 1000-grain weight, dispersed across the genomes, and located within previously mapped QTL	Boyles et al., 2016
1315 accessions; 36,285 SNPs	101 SNPs associated with at least one of the 9 plant architecture traits; *KS3* and *GA2ox5* associated with seed number and plant height, respectively; novel QTL for tillers, stem circumference, internode number, seed number, panicle exsertion, and length	Zhao J. et al., 2016
1,000 accessions; 265,000 SNPs	SNPs with distinct haplotypes confer variation in plant height and inflorescence architecture traits	Morris et al., 2013
**Bread wheat (*Triticum aestivum*)**		
210 winter wheat accessions; 7.928 SNPs	Novel QTL and candidate genes reported that are involved in assimilate partitioning, floret fertility, spike morphology and grain numbers	Guo et al., 2016
130 elite lines and landraces; 90K SNP array	5 and 32 SNPs for spike ethylene, and 22 and 42 SNP for spike dry weight, in glasshouse and field conditions, respectively; some SNPs closely localized to SNPs for plant height, suggesting close association between plant height and spike related traits	Valluru et al., 2017

A large GWAS study in rice detected 42 significant genotype–phenotype associations for plant morphology, grain quality, and root architecture traits, which in most cases were co-localized with QTL and candidate genes controlling the phenotypic variation of single or multiple traits (Biscarini et al., 2016). Several SNPs in rice were associated with plant and panicle architecture, biomass and yield (Zhao et al., 2011; Yang et al., 2014; Ya-fang et al., 2015; Rebolledo et al., 2016), while candidate genes in pathways regulating plant architecture overlap with QTL associated with panicle architecture traits (Crowell et al., 2016; Rebolledo et al., 2016).

In wheat, candidate genes associated with SNPs were involved in carbohydrate metabolism, floral fertility, spike morphology and grain number, providing valuable targets for selection (Guo et al., 2016). Significant marker-trait associations also provided insight into genetic architecture of flowering, plant height and grain weight in barley (Pasam et al., 2012). Individual QTL accounted, however, only for a small portion of phenotypic variation.

In sorghum, several SNPs were associated with plant and inflorescence architecture traits, with many located within previously mapped QTL (Morris et al., 2013; Maurer et al., 2015; Boyles et al., 2016; Zhao J. et al., 2016). Candidate genes *KS3* (associated with seed number) and *GA2ox5* (associated with plant height) were also reported (Zhao J. et al., 2016). A QTL with a major effect corresponded to the priori known photoperiod response gene *Ppd-H1* (Maurer et al., 2015).

#### Abiotic Stress Adaptation in Soybean

Multiple SNPs are reported to be associated with tolerance to drought and heat stress in soybean (**Table 5**). Dhanapal et al. (2015) reported 39 SNPs associated with carbon isotope ratio (δ^13^C), which is a surrogate trait to measure water use efficiency. The genomic distribution of these SNPs revealed that several are co-located and likely tag the same locus, suggesting that markers for δ^13^C can be identified in soybean using GWAS. Dhanapal et al. (2016) reported 52 unique SNPs for total chlorophyll content tagged on 27 loci across 16 chromosomes. While many of these putative loci were near genes previously identified or annotated as related to chlorophyll traits (Hao et al., 2012), numerous SNPs marked chromosomal regions with unknown-function genes.

**Table 5:** SNPs and germplasm-based GWAS for abiotic stress tolerance in soybean (*Glycine max*).

Association mapping panel and SNPs	Summary of marker-trait association and candidate genes identified	Reference
373 accessions; 31,145 SNPs	31 SNPs, associated with photochemical reflectance index and measure of non-photochemical quenching, tagged into 15 putative loci on 11 chromosomes	Herritt et al., 2016
219 accessions; 1536 SNPs	19 SNPs associated with low P-tolerance QTL, with a novel cluster of SNPs on chromosome 3 associated with more than one trait	Ning et al., 2016
332 accessions; 31,253 SNPs	52 unique SNPs tagged in 27 putative loci associated with total chlorophyll content	Dhanapal et al., 2016
373 accessions; 12,347 SNPs	39 SNPs, tagged at 21 loci, associated with carbon isotope ratio (δ^13^C), 15 of these located within a gene	Dhanapal et al., 2015

Non-photochemical quenching (NPQ) under abiotic stress conditions protects plants from heat when more light is absorbed than can be used for photosynthesis (Li et al., 2009). Canopy reflectance measured as photochemical reflectance index (PRI), amenable for high throughput field phenotyping, is a surrogate to measure NPQ (Gamon et al., 1992). Thirty-one PRI-specific SNPs, tagged in 15 loci on 11 chromosome harboring candidate genes associated with NPQ, photosynthesis, and sugar transport, may provide an opportunity to improve photosynthesis in soybean (Herritt et al., 2016).

#### Abiotic Stress Adaptation in Cereals

Cereal crops have been extensively investigated for SNPs and candidate genes associated with abiotic stress adaptation (**Table 6**). Ethylene levels have been linked to yield penalty under heat stress in wheat, largely due to reduction in spike fertility and grain weight (Hays et al., 2007). Valluru et al. (2017) reported 5 and 32 significant SNPs associated with spike ethylene, and 22 and 142 significant SNPs associated with spike dry weight, in greenhouse and field studies, respectively. Some of these SNPs are close to SNPs associated with plant height, suggesting associations between plant height and spike-related traits. This opens the possibility of gene discovery and breeding of wheat cultivars with reduced ethylene effects on yield under heat. The D genome progenitor of bread wheat *Aegilops tauschii* has potential as an excellent source of abiotic stress tolerance. Qin et al. (2016) reported 25 SNPs and several putative candidate genes (enzyme, storage protein, and drought-induced protein) associated with drought adaptation, while Liu Y. et al. (2015) found 13 SNPs and putative candidate genes related to P-deficiency tolerance.

**Table 6:** SNPs and germplasm-based GWAS for abiotic stress tolerance in barley, pearl millet, rice, and sorghum.

Association mapping panel and SNPs	Summary of marker-trait association and candidate genes identified	Reference
**Barley (*Hordeum vulgare*)**		
179 accessions; 5,892 SNPs	17 QTL for root/shoot traits, with exotic alleles at 14 loci; a QTL on chromosome 1H accounted for root dry weight and tiller number; exotic alleles at 7 loci significantly interacted with drought stress	Reinert et al., 2016
167 accessions; 7,864 SNPs	60 significant marker-trait associations; grain yield under heat stress on 2H, yield stability on 7H and grain yield under elevated CO_2_ on 4 H and 7H under two factor treatments, while markers from single factor were not retrieved under two factor treatments	Ingvordsen et al., 2015
**Pearl millet (*Pennisetum glaucum*)**		
250 inbreds; 46 SNPs and InDels from 17 genes of a known drought tolerant QTL	7 SNPs from five genes common under varying moisture stress; a SNP associated with grain yield and harvest index, while a InDel with stay-green and yield under drought stress	Sehgal et al., 2015a
**Rice (*Oryza sativa*)**		
391 temperate rice accessions; 57,000 SNPs	31 significant genotype-phenotype associations detected: 21 and 10 for plant and root architecture traits, respectively, and colocalized with QTL and candidate gene traits controlling phenotypic variation	Biscarini et al., 2016
220 accessions; 6,000 SNPs	20 and 44 SNPs, respectively, associated with Na^+^/K^+^ ratio and grain yield under stress contributed 5-18% phenotypic variance; the region harboring *Saltol*, a major QTL on chromosome 1, associated with Na^+^/K^+^ ratio; SNPs representing new QTL on chromosome 4, 6, 7	Kumar et al., 2015
292 accessions; 44K SNPs array	SNPs associated with phosphorus use efficiency (PUE) on chromosomes 1, 4, 11 and 12, with distinct haplotypes contributed greatest PUE	Wissuwa et al., 2015
413 accessions; 44,000 SNPs	Four regions co-localized with *a priori* candidate genes for Al tolerance, while two regions co-localized with previously identified QTL	Famoso et al., 2011
**Sorghum (*Sorghum bicolor*)**		
343 accessions; 325,487 SNPs	14 SNPs with two heat stress responsive traits, leaf firing and blotching, with many candidate genes near SNPs linked to biological pathways involved in plant stress responses including heat stress	Chen et al., 2017
1943 landraces; 404,627 SNPs	Genic SNPs associated with environment variables predicted genotype × interactions under drought stress	Lasky et al., 2015
187 accessions; 220 934 SNPs	A major Al-tolerance gene, *SbMATE*, collocated in a genomic region on chromosome 3 associated with grain yield, and *SbMATE* specific SNPs under –P conditions showed very high associations to grain yield production, contributed up to 16% variation	Leiser et al., 2014
**Bread wheat (*Triticum aestivum*)**		
373 *A. tauschii* accessions; 7,185 SNPs	25 SNPs associated with traits related to drought resistance; several candidate/flanking genes associated with drought resistance grouped into three categories per the type of encoded protein (enzyme, storage protein, and drought-induced protein)	Qin et al., 2016
380 *A. tauschii* accessions; 7,185 SNPs	13 SNPs associated with P-deficiency tolerance traits distributed on six of the seven *A. tauschii* chromosomes; several candidate/flanking genes related to P-deficiency tolerance grouped in five categories by the types of proteins they encoded (defense response proteins, enzymes, promoters and transcription factors, storage proteins, or proteins triggered by P deficiency)	Liu Y. et al., 2015

A major Al-tolerance gene *SbMATE* on chromosome 3 has been shown to be associated with grain yield in sorghum, where *SbMATE* specific SNPs under –P conditions contributed up to 16% genotypic variance (Leiser et al., 2014). Forty-eight genomic regions associated with Al tolerance were reported in rice, four of which co-localized with *a priori* known candidate genes, and two co-located with previously identified QTL (Famoso et al., 2011).

In barley, a genomic region on chromosome 2H was associated with grain yield under heat stress, a region on chr 7H with grain yield, and a region on chr 4H and chr 7H with elevated CO_2_ under two factor treatments (high temperature and elevated CO_2_). None of the SNPs associated with single factor treatments were retrieved under two factor treatments, thus emphasizing the importance of multifactor treatments (Ingvordsen et al., 2015).

Genic SNPs associated with environmental variations (but independent of geographical location) predicted genotype × environment interactions for drought stress and aluminum toxicity in sorghum (Lasky et al., 2015). Wissuwa et al. (2015) reported several SNP loci associated with phosphorus use efficiency (PUE) in rice on chromosomes 1, 4, 11, and 12. A minor *indica*-specific haplotype on chromosome 1 and a rare *aus*-specific haplotype on chromosome 11 displayed the highest PUE, and could have potential for targeted introgression while breeding for rice under P-limited cropping systems.

Emerging evidence suggests that responses to stress combinations cannot be reliably predicted from the responses to individual stresses (Makumburage et al., 2013). An integrated approach is therefore needed to model the genetics of responses to a range of single and combined stresses. For example, association analysis report QTL with contrasting and with similar responses to biotic versus abiotic stresses, and below-ground versus above-ground stresses. There is a need to conduct multi-trait GWAS to identify robust candidate genes for multiple stresses (Thoen et al., 2016).

The proliferation of genome wide association analyses has led to identification of candidate loci (often co-located with major QTLs or candidate genes) associated with abiotic stress adaptation, phenology and plant architecture, and edible yield. The identification of such loci can facilitate genomics-assisted breeding in cereal and legumes.

### TILLING: Mutagenesis and Reverse Genetics for Elucidating Gene Function

Chemical mutagenesis and subsequent screening for mutations linked to altered agronomic phenotypes is a reverse genetic technique to identify candidate genes for crop improvement. Targeting Induced Local Lesions IN Genomics (TILLING) is the commonly used approach, employing a mismatch-specific endonuclease to detect single base pair (bp) allelic variation in a target gene (Gilchrist and Haughn, 2005). TILLING by sequencing (Tsai et al., 2011) can greatly increase throughput and novel allele discovery by applying second-generation sequencing approaches rather than endonucleases to facilitate variant discovery across the genome rather than in individual genes (Henry et al., 2014; Kumar et al., 2017). TILLING has been successfully used to detect both induced and natural variations in a wide range of plant species, including: novel allelic variation in the barley genes *HvCO1*, *Rpg1*, *elF4E*, *HvHox1*, *BMY1*, *GBSS1*, *LDA1*, *SSI*, *SSlla, mlo and Mla* (Mejlhede et al., 2006; Talamé et al., 2008; Gottwald et al., 2009; Sparla et al., 2014); the maize genes *DMT101*, *DMT102*, *DMT103*, *DMT 106*, *HAC110*, *HDA105* (Till et al., 2004); and the wheat genes *PpD-1*, *Rubisco activase A* and *Rubisco activase B* (Chen et al., 2012). In sorghum, TILLING generated a functional-effect point mutation in the *CYP79A1* gene, generating sorghum lines with reduced levels of the cyanogenic glycoside dhurrin, which has potential to enhance the use of this widely grown crop as forage for livestock (Blomstedt et al., 2012). A TILLING-induced mutation in a *TI1* protease inhibitor increased the digestibility and thus nutritional value of pea. Although mutagenesis in TILLING approaches is untargeted and does not provide the versatility of genome editing, crops improved using chemical or radiation mutagenesis via TILLING are not regulated as GMOs in most jurisdictions, increasing their commercial competitiveness with more precise genome editing approaches (Kumar et al., 2017).

### Using Haplotypes to Identify Alleles in Cultigen Pools

A haplotype is a combination of DNA polymorphisms (markers, alleles) that are tightly linked to each other on a chromosome and hence tend to be inherited together from parent to offspring. Maize was among the first crops for which a comprehensive haplotype map was generated, which showed highly divergent haplotypes and recombination rates based on several million sequence polymorphisms in 27 diverse inbred lines (Gore et al., 2009). This research also identified hundreds of selective sweeps and highly distinct chromosome regions likely bearing loci related to domestication and geographic adaptation. Genetic structure and subpopulation structure are also associated with origin of germplasm and post-domestication selection, as revealed by comparative haplotype analysis in tropical and temperate maize germplasm (Lu et al., 2011). Moreover, Thirunavukkarasu et al. (2017) were able to identify 252 haplotype blocks in subtropical elite inbred maize lines, which varied in size from 1 to 15.8 Mb, with slow LD decay (200–300 Kb) across all chromosomes, suggesting selection of favorable traits around low LD regions in breeding programs. Due to strong population substructure, this subtropical maize germplasm grouped into three distinct clusters, which provides means for exploiting heterotic potential among them. The use of haplotypes improved mapping efficiency to detect QTL related to drought adaptation in maize (Lu et al., 2009). Furthermore, integrated mapping (based on independent linkage and LD analysis) along with haplotypes led to identification of significant QTL explaining up to ca. 35% of phenotypic variation. Two significant haplotypes were involved in the control of flowering time, and encoding aldo-keto reductases associated with detoxification pathways contributing to cellular damage due to stress.

There is a continual need to identify allelic variants conferring desirable agronomic traits. For example, recent haplotype analysis in Indian wild rice identified the variants H5 and H1 of *HKT1;5* and *HKT2;3* as associated with high salinity tolerance (Mishra et al., 2016b). Haplotype variation of major and few minor alleles seems to be distributed over distant geographic regions (Mishra et al., 2016a). Such alleles may be useful for broadening the range of cultivars to enhance rice productivity in salt-prone areas. The rice DNA markers RM 464A and RM 219 at the *Sub-1* locus of chromosome 9 (which accounts for 70% of phenotypic variation for submergence tolerance) have assisted in breeding cultivars that are tolerant to submergence for up to 2 weeks during the vegetative growth stage (Rathnayake et al., 2012). The *Sub-1* locus encodes the *ethylene-responsive factor* (*ERF*) genes *sub1B* (from the submergence tolerant FR13A landrace) and *Sub1C* in all *Oryza sativa* cultivars, while the *ERF* paralog *Sub1A* is found in a subset of *O*. *sativa* ssp. *indica* accessions, and seems to arise from duplication of *Sub1B* (Fukao et al., 2009). Some submergence tolerant rice accessions lack *Sub1A* (Tamang et al., 2011), which appears to suppress leaf elongation under submergence (Singh et al., 2010). This suggests that *Sub1A* may not be the only contributing factor to submergence tolerance in rice (Samal et al., 2014).

The haplotype map of disomic hexaploid bread wheat, based on resequencing 62 wheat lines using exome capturing and genotype-by-sequencing, has exposed distinct patterns of directional selection in homeologous genomes (Jordan et al., 2015). This finding suggests that the likelihood of beneficial allele recovery was increased in bread wheat by broadening the set of selection targets. Haplotype analysis of stem rust resistance genes revealed that most breeding lines (83 out of 115) released by CIMMYT until the 2000s carry *Sr2.* Five were found to carry the *Sr25* haplotype, while a small number of (5 out of 22) cultivars bred by the United States Department of Agriculture haboured the *Sr2*, *Sr24*, *Sr36* haplotypes. *Sr2* was also found in two out of 43 wheat breeding lines from China (Yu et al., 2010). Diverse bread wheat lines bred in different Africa countries have been found to harbor the *Sr2, Sr36, Sr24*, *Sr31*and *Lr34/Yr18/Sr57* haplotypes (Prins et al., 2016). Tetraploid Ethiopian durum wheat landraces and bred cultivars carry the *Sr2* and *Sr22* haplotypes, with only a few bearing *Sr13* (Haile et al., 2013). Haplotype analysis also located the origin of *Sr33*, an ortholog of a barley mildew resistance *Mla* gene that was introgressed to bread wheat from the wild relative *Aegilops tauschii* (Periyannan et al., 2013). Such analyses can identify sources of novel alleles for use in improving host plant resistance through breeding.

### Landraces and Wild Relatives

Landraces are a repository of crop genetic diversity that have evolved through natural and artificial selection over millennia, and represent valuable resources for crop adaptation to stresses. For example, the allelic variation amongst rice and wheat landraces has provided agronomically beneficial traits for abiotic stress tolerance (Dwivedi et al., 2016). Pasam et al. (2014) noted that widely adapted (5°–62.5° N, 16°–71° E) spring barley landraces (L_RC_1485), which showed abundant genetic diversity, clustered into six major germplasm groups, differentiated by geographical origin and latitude, ear row type, caryopsis types, and climate zones. Creole wheat landraces introduced into Mexico from Europe are adapted to a wide range of climatic regimes and represent a useful genetic resource. Vikram et al. (2016) characterized 9,416 landrace accessions using genotyping-by-sequencing and identified 15 genetic groups that are likely adapted to specific environments of Mexico, with some groups adapted to extreme environments. For example, landraces from Michoacán (high temperature and rainfall) and Durango (high annual average temperature and low precipitation) had an exceptionally high frequency of rare alleles, which may be a contributing factor of landrace adaptation to these climates. A similar study on local adaptation of barley landraces in Ethiopia revealed that environmental differences (temperature and precipitation) and geographic effects contributed 40 and 29% of the explained genetic variation, respectively (Abebe et al., 2015). Pearl millet landraces (249 accessions) from Senegal were genetically distinct from many global accessions^1^, 262 accessions from Africa, Asia, and the American contents, with the greatest representation from India, Kenya, South Africa, Yemen, and Zimbabwe, showed little population structure, and higher-levels of linkage disequilibrium decay, providing a valuable resource for use in breeding (Hu et al., 2015). Population structure analysis involving cowpea landraces and wild relatives delineated most African landraces into two major genepools, with most landraces from West Africa forming genepool 1, while the majority of the landraces in genepool 2 were from East Africa. Furthermore, the authors noted that each genepool was closely related to wild cowpea in the same geographic region, suggesting divergent domestication leading to the formation of two genepools in cowpea (Huynh et al., 2013). Lentil (*Lens culinaris*) landraces (predominantly from Greece and Turkey) also have revealed high levels of genetic diversity (Lombardi et al., 2014).

Wild and weedy relatives of crops are an important source of adaptation and stress tolerance genes. Wild species often grow in harsh environments and therefore could be the source of genes conferring abiotic stress adaptation. The greatest impact of wild relatives in crop improvement to date have been in increasing host plant resistance to pathogens and pests in several crops. Wild species have also been the source of genes for edible yield and quality traits in some crops (Dwivedi et al., 2008). Poets et al. (2015) compared SNP polymorphism between landraces and wild barley accessions and noted that landraces comprised multiple source populations with unequivocal contributions from wild barley populations across the genome. Furthermore, two genomic regions on the 2H and 5H chromosomes contributed to geographic differentiation in allele frequencies (Fang et al., 2014). Wild barley accessions collected at ‘Evolution Canyon’ at Nahal Oren, Israel were more genetically diverse than those from other regions in northern Israel, while those from the hot and dry south-facing slope were genetically more distinct from north-facing slope accessions (Bedada et al., 2014).

A study on genetic basis of phenotypic variations among wild pearl millet populations from two north–south aridity gradients in West Africa revealed that the size of the inflorescence, the number of flowers and above-ground dry mass co-varied positively with rainfall decrease. Moreover, two SNPs located in the *Myosin XI* gene were significantly associated with variation in the average flower number. Both the allele frequency of the two SNPs and the average flower number co-varied with the rainfall gradient on the two gradients. *Myosin XI* is a good candidate for fitness-related adaptation in wild populations (Ousseini et al., 2017). Structure analysis of 99 ecotypes of wild soybean, sampled across their native geographic range and genotyped by SoySNP50K array, identified four genetic groups that largely corresponded to geographic regions of central China, northern China, Korea, and Japan, with high levels of admixture between genetic groups. Moreover, the environmental factors contributed 23.6% to population differentiation, while geographical factors accounted for 6.6%. Precipitation variables explained divergence of the groups along longitudinal axes, whereas temperature variables contributed more to latitudinal divergence (Leamy et al., 2016).

Such delineation of landraces and wild relatives into groups of genetic relatedness associated with geographic or environmental differences, and identification of accessions harboring higher numbers of rare alleles (with functional effects) will be valuable genetic resources in breeding and for improving the management and utilization of germplasm in crop improvement. There are significant barriers (both pre-fertilization and post-fertilization) to inter-specific hybridization. Technology for circumventing these barriers are required for increased introgression of allelic variation from wild relatives to primary crop genepools (Dwivedi et al., 2008).

## Key Breeding Targets to Enhance Adaptation and Productivity

### Optimizing Crop Productivity Using Mutagenesis in the Florigen Pathway

Flowering time, the transition from vegetative to reproductive growth, is a major determinant of crop yield (Cockram et al., 2007; Jung and Muller, 2009). The universal flowering activator florigen has several genetic components, including the key *FLOWERING LOCUS T* (*FT*) gene (Koornneef et al., 1991; Samach et al., 2000; Turck et al., 2008). While *FT* and *FT*-like genes generally activate flowering, another group of genes including *TERMINAL FLOWER 1* (*TFL1*) and *TFL1*-like genes act as flowering repressors (Karlgren et al., 2011). These genes belong to the CENTRORADIALIS/TERMINAL FLOWER 1/SELF-PRUNING (CETS) gene family, which display sequence similarity to the phosphatidylethanolamine binding protein (PEBP) genes. The balance and interplay between flowering activators and repressors determines flowering response. Selection of variant genes in the florigen pathway to increase environmental flexibility and yield has played in important role in the domestication and improvement of many crops, including barley (Comadran et al., 2012) and rice (Ogiso-Tanaka et al., 2013).

Recently, more targeted breeding approaches have used florigen pathway genes to control flowering in crops. A study in tomato showed that reproductive growth could be influenced by combining mutations in *SINGLE FLOWER TRUSS (SFT;* an FT homolog) and a bZIP transcription factor within the florigen pathway, to produce plant architecture increasing yields (Park et al., 2014). Similarly, *SFT* heterozygosity in tomato was also observed to alter plant architecture (Jiang K. et al., 2013). Using the CRISPR/Cas9 system in tomato, it has been demonstrated that mutation of the floral repressor gene *SP5* can produce early yielding plants (Soyk et al., 2017).

Rational engineering of flowering time remains constrained by a lack of knowledge on the functions of many components of the florigen pathway in different plant species, which contains many closely related genes with diverse functions, including unknown interaction and regulation networks. Because of the diversification of the florigen pathway genes in flowering plants, an improved understanding of species-specific florigen pathways will be important for crop breeding (Zhang et al., 2010). Recent studies investigating genetic control of flowering have identified an important regulator of florigen transport in rice (Song et al., 2017). Other studies have identified a loss of vernalization requirement in narrow-leafed lupin (*Lupinus albus*), caused by a deletion in the *FT* promoter (Nelson et al., 2017). In *Arabidopsis thaliana*, mutagenesis of codons within the *FT* gene identified differences critical for the related antagonist TFL1 and indicated potential candidate transcription factors interacting with the protein. As knowledge of species-specific flowering mechanisms develops, fine-tuning of the florigen pathway should allow yield increases through better control of flowering and growth in crops.

### Enhancing Abiotic Stress Adaptation

Breeding for adaptation to abiotic-stress remains a challenging task (Dwivedi et al., 2010, 2017; Kole et al., 2015). Conventional crossing and selection for abiotic stress adaptation had had limited success. However, when supported by applied genomics tools and genetic engineering, accelerated introgression of beneficial alleles has enhanced yield and abiotic stress adaptation in cereals and legumes.

Six large-effect QTL related to drought adaptation have been shown to be effective in multiple genetic backgrounds and production environments in rice. Pyramiding of these large-effect QTL has improved drought adaptation of widely grown Asian cultivars (Kumar et al., 2014). Submergence tolerant rice cultivars bearing *SUB1A-1* have had significant impacts in Asia (Bailey-Serres et al., 2010; Dar et al., 2013). Furthermore, pyramiding *SUB1A-1* and drought-tolerant QTL (Kumar et al., 2014) or salt (*Saltol1*) and flood (*Sub1*) tolerance QTL (Mackill et al., 2012) is expected to lead to improved cultivars adapted to stress-prone lands in Asia. The *Pup1* allele increases P uptake and confers significant grain yield advantage in rice grown on P-deficient soils (Wissuwa et al., 2002). Introgressed lines containing *Pup1* significantly increased grain yield on P-deficient soils (Chin et al., 2011). Also, overexpression of a *Pup1-specific protein kinase gene* (*PSTOL1*) significantly enhances grain yield in P-deficient soils. *POSTL1* promotes early root growth, thereby enabling plants to acquire more P and other nutrients (Gamuyao et al., 2012).

Identifying SNPs that are robustly associated with environmental adaptation can be useful for crop improvement. Environment-associated SNPs independent of geographic origins predicted genotype × environment interactions under drought stress and aluminum toxicity in sorghum (Lasky et al., 2015). Climatic variables accounted for most of the genetic variation in barley (Abebe et al., 2015). Many SNPs were associated with putative adaptive loci and candidate genes conferring enhanced adaptation. In barley, five and two SNPs were correlated with length of growth season and precipitation, respectively (Selçuk et al., 2015). However, none were correlated with both. More recently, Russell et al. (2016) noted extensive sequence variations amongst known flowering associated barley genes *HvCEN*, *HvPPD*, and *HvFT1* (Turner et al., 2005; Casas et al., 2011; Comadran et al., 2012), with haplotypes exhibiting strong geographical structuring, likely contributed to range-wide ecogeographical adaptation. In maize, Westengen et al. (2012) noted that 79 and 22 SNPs associated with maximum temperature and mean precipitation, respectively, with many located in genes functioning in abiotic stress adaptation. Non-synonymous SNPs clustered in the region harboring six known QTL associated with relatively high phenotypic variation for drought adaptation in maize (Xu et al., 2014). Pyhäjärvi et al. (2013) observed that SNPs in wild teosinte ancestor were associated with altitude. Millet et al. (2016) noted 8 and 12 QTL associated with heat and drought stress adaptation in maize, respectively, with low or negative effects in favorable environments. Another 24 QTL improved yield in favorable environments but without any effects under stress, thereby indicating that QTL effects were expressed as functions of environmental variables and scenarios. Such genomics-assisted knowledge can potentially be used to accelerate breeding for drought-prone environments (Millet et al., 2016).

African pearl millet landraces collected in 2003 displayed a short life cycle, reduced plant and spike size, and increased frequency of early flowering alleles (from 9.9% in 1976 to 18.3% in 2003) at the flowering locus *PHYC* (Saïdou et al., 2009), which suggests that recurrent drought may promote shortening of growth duration in pearl millet (Vigouroux et al., 2011). Similar observations were noted after monitoring changes in functional diversity due to possible climate effects in wild emmer wheat and wild barley populations. Populations collected in 2008 flowered earlier than those collected in 1980, with greater shortening of flowering time after 28 years for wild barley than wild emmer wheat. However, the study indicated that emmer wheat lost more alleles than wild barley. The allelic reduction in emmer wheat was negatively correlated with altitude (-0.854^∗^) and humidity (-0.673^∗^), while in barley the difference between the sampling years was positively correlated with rainfall (0.790^∗^) but negatively with evaporation (-0.692^∗^) (Nevo et al., 2012).

Villordo-Pineda et al. (2015) have identified 37 SNPs with a potential drought adaptation function in common bean. A ‘QTL-hotspot’ region harboring 12 QTL associated with drought adaptation traits contributed up to 58% of the phenotypic variation in chickpea (Varshney et al., 2014). Indeed, introgressions containing this region in JG 11, a widely grown cultivar in India, have improved root traits and drought tolerance (Varshney et al., 2013). Subsequently, Kale et al. (2015) fine-mapped this ‘QTL-hotspot’ and identified four candidate genes from this region that are associated with drought tolerance. Anderson et al. (2016) identified several candidate loci that putatively contributed to adaptation to abiotic stresses, which may permit targeted use of *Glycine soja* germplasm for enhancing the genetic potential of cultivated soybeans. Qi et al. (2014) noted that sequence variations in *GmCHX1* were associated with salt tolerance in a wild soybean, W05. Likewise, 20 loci associated with P efficiency-related traits have been identified in soybean, some coinciding with known P efficiency-related genes *GmACP1* and *GmPT1*, while *Glyma.04G214000* and *Glyma.13G161900* displayed differential expression in low-P soils (Zhang et al., 2016c).

The evidence to date suggests that understanding (and deployment) of major QTL or candidate genes associated with abiotic stress adaptation has led to the development and release of several maize and rice cultivars adapted to different abiotic stresses (Ortiz, 2013). A large effort is underway to introgress major QTL associated with drought and heat stress adaptation in common bean and chickpea (Dwivedi et al., 2017). The discovery of several SNPs associated with variation in both temperature and precipitation responses in barley, maize and sorghum provide a further opportunity to develop cultivars with enhanced fitness in the context of a changing climate.

### Phenology, Yield and Adaptation

#### Crop Duration and Yield

Understanding the nucleotide variation and mechanism of molecular evolution of flowering, maturity and plant height genes could accelerate the development of cultivars of specific duration to better adapt them to growing seasons. Knowledge on sequence variation in genes related to plant or panicle architecture (and yield) may provide opportunities to genetically enhance crop productivity *per se*.

Rice adaptation to climate is influenced by days to flowering and its sensitivity to photoperiod variation. *OsPRR37* (*PRR37*) is within the *Early heading 7-2* (*EH7-2*)/*Heading date 2* (*Hd2*) QTL in rice. The *japonica* cultivars having *Ghd7/Hd4* and *PRR37/Hd2* non-functional alleles flower early under extended photoperiod, and are adapted to the northernmost region of cultivation, up to 53°N latitude. Genetic analysis reveals that the effects *PRR37* and *Ghd7* effects on heading date are additive (Koo et al., 2013). *PRR37* down-regulates *Hd3a* expression to suppress flowering under extended photoperiods, thus suggesting that *PRR37/Hd2* and *Ghd7/Hd4* contributed to adaptation of rice in temperate and cool regions. Further investigation using accessions from the *O. japonica* core collection have revealed that *RICE FLOWERING LOCUS T1* (*RFT1*) is the major contributor to flowering among *japonica* cultivars adapted to northern areas (Naranjo et al., 2014). *Ghd7* is a gene with pleotropic effects that controls plant height, heading date and yield in rice. Lu et al. (2012) noted 76 SNPs and six indels within a 3932bp DNA fragment of *Ghd7* derived from two distinct ancestral genepools (*indica* and *japonica*), of which SNP S_55 was associated with plant height while another seven SNPs were in complete linkage with spikelets per panicle, regardless of photoperiod. Their finding suggests major flexibility of *Ghd7* to improving phenology, panicle architecture, and yield in rice.

The genes *IDEAL PLANT ARCHITECTURE* (*IPA*), *LONG PANICLE1* (*LP1*), *SPIKELET NUMBER* (*SPIKE), Gna1* (grain number), *Ghd7* (grain number, plant height, and flowering), *GS3* (grain weight and length), *GW5* (grain weight), and *DEP1* (*DENSE AND ERECT PANICLE1*) greatly influence panicle architecture and seed yield in rice (Ashikari et al., 2005; Fan et al., 2006; Weng et al., 2008; Xue et al., 2008; Huang et al., 2009; Jiao et al., 2010; Miura et al., 2010; Fujita et al., 2013; Liu E. et al., 2016). *DEP1* locus has been widely used for developing high yield rice cultivars with erect panicle architecture (Yan et al., 2013). Mining allelic variations for panicle traits unravels 45 SNPs and 26 InDels within the DNA fragment of *DEP1* and replacement of 637 bp by 12 bp fragment explain most of the phenotypic variations for panicle architecture, and SNP(G/C) largely affects branches and grains panicle^-1^ (Zhao M. et al., 2016).

*Ghd7* (*Ma6*) and *pseudoresponse regulator protein 37* (*PRR37*) alleles in sorghum confer differences in photoperiod sensitivity and flowering times that are critical for production of high-biomass energy or grain sorghum (Murphy et al., 2011, 2014). Furthermore, Wang Y. et al. (2014) investigated nucleotide diversity of *Ma3*, another maturity gene in sorghum, and identified three and 17 SNPs that affected flowering at high-latitude and at low-latitude environments, respectively. Indeed, a major QTL on chromosome 6, *FlrAvgD1*, which contributed 85.7% of variation in flowering under LD, was narrowed to a 10 kb interval containing the only one annotated protein-coding gene (*Sb06g012260*) with potential to accelerate cross-utilization of temperate and tropical germplasm for production of grain or bioenergy sorghum types (Cuevas et al., 2016).

The discovery and deployment of alleles of semi-dwarfing genes have contributed to enhanced lodging resistance and increased productivity in both rice and wheat (Peng et al., 1999). For example, the use of *semi-dwarf1* (*sd1*) in rice and *reduced height* (*Rht*) alleles in wheat, which encode a GA biosynthesis enzyme and a dominant suppressor protein of GA signal transduction, respectively, have been widely used to confer lodging resistance in these crops (Peng et al., 1999; Sasaki et al., 2002). A semi-dwarfing gene (*sdw1)* locus has been widely introgressed into barley cultivars grown worldwide. At least four alleles (*sdw1.a*, *sdw1.c*, *sdw1.d*, and *sdw1.e*) have been reported (Franckowiak and Lundqvist, 2012). The *gibberellin 20-oxidase* gene (*HvGA20ox2*) is the functional gene of *sdw1* mutants, and deletions resulted in different functional alleles for breeding purposes. Diagnostic markers can differentiate the wild type allele from the *sdw1.d*, *sdw1.a*, and *sdw1.c* alleles (Xu Y. et al., 2017). In sorghum, four unlinked dwarfing genes (*Dw1–Dw4*) were combined to reduce plant height to increase lodging resistance and improve mechanized harvesting (Quinby and Karper, 1954). Of these, only *Dw3* has been cloned (Multani et al., 2003). Yamaguchi et al. (2016) isolated the *Dw1* gene, which encodes a novel uncharacterized protein. A histological analysis comparing the Near Isogenic Line (NIL)-*dw1* with that of wild type showed similar longitudinal parenchymal cell lengths of the internode, but significantly reduced number of cells per internode in NIL-*dw1*. NILs containing *dw1* and *dw3* displayed a synergistic phenotype, which contributes to improved lodging resistance and mechanical harvesting in sorghum (Yamaguchi et al., 2016).

To date, 10 major genes (*E1* to *E9* and *J*) and several QTL have been shown to be involved in control of flowering in soybean. Different allele combinations at *E1*-*E4* and *E9* loci produce diverse flowering habits in soybean cultivars (Xu et al., 2013; Kong et al., 2014; Tsubokura et al., 2014; Zhao C. et al., 2016). Multi-locus genotypes involving *E1* to *E4* account for 62–66% of natural variation in the flowering time and identified a new allele in *E1* locus, *e1-re*, for flowering in soybean (Tsubokura et al., 2014). *FT2a* and *FT5a*, the orthologs of *FLOWERING LOCUS T* (*FT*) (Kong et al., 2010), play a major role in initiation of flowering. Indeed, their expression in response to photoperiod is controlled by different allelic combinations involving *E1* to *E4*. More recently, Takeshima et al. (2016) identified a QTL in LG J which was localized to a genomic region of 107 kb (harboring *FT5a*). The study detected SNP polymorphisms between the parents involving early (*ef*) and late (*lf*) flowering alleles, and also detected *ef*, a rare haplotype distinct from others including *lf*. A higher transcript abundance of *FT5a* in NILs containing *ef* allele suggests that differential transcriptional activities or mRNA stability may cause differences in flowering (Takeshima et al., 2016).

An investigation of flowering time variation and SNP polymorphisms in key regulatory genes in common bean revealed that *PvVRN1* and *Pv PHYB* are associated with days to flowering, *PvMYB29* with number of flower buds per inflorescence, and *PvTFL1z* and *PvFCA* with inflorescence length (Raggi et al., 2014). More recently, a QTL on linkage group Pv01, harboring the *Phvul.001G189200* gene (with sequence similarity to the *TERMINALFLOWER1* (*TFL1*) gene in *Arabidopsis thaliana*), explained up to 32% of phenotypic variation for time to flowering, 66% for vegetative growth, and 19% for rate of plant production, supporting *Phvul.001G189200* (referred as *PvTFL1y*) as a candidate gene for determinacy locus in common bean (González et al., 2016). *CcTFL1*, a candidate gene for determinacy in pigeonpea, contributed substantial phenotypic variations for determinacy (45–96%), flowering (45%) and plant height (77%) (Mir et al., 2014). Foucher et al. (2003) isolated three *TFL1* homologs, *PsTFL1a*, *PsTFL1b*, and *PsTFL1c* in pea. *PsTFL1a* controls indeterminacy of the apical meristem during flowering, while *PsTFL1c* delays the induction of flowering by lengthening the vegetative phase in pea. The development of genetic markers has potential to allow manipulation of the determinacy trait in these and other legume species.

Understanding the molecular basis of allelic variation associated with flowering, plant height, maturity, plant architecture, and yield provides opportunities to tailor crop ideotypes that are better adapted to specific agro-ecosystems or meeting end-use preferences.

#### Tropical vs. Temperate Adaptation

Although maize, rice and sorghum were domesticated in tropical regions, they are all commercially grown both in tropical and temperate climates. Understanding the molecular basis of such adaptation differences for these important cereal crops is critical for targeted introgression of beneficial alleles from one genepool to another, or for developing grain or bioenergy sorghum types. Sorghum is a short-day plant requiring a daylength below 12 h 20 min to induce flowering. Hence, most of the tropical sorghum germplasm flowers too late or is too tall to be exploited for seed production in temperate environments. In the 1970s a large-scale sorghum conversion program was initiated by USDA-ARS to convert tropical accessions to plants adapted to temperate zones by introgressing recessive day-neutral flowering alleles and dwarf-height genes into the exotic backgrounds via a backcross scheme to recover the exotic genome in early flowering, combine-height inbred lines (Stephens et al., 1967). This effort resulted in the release of 40 such converted lines for use in temperate zones worldwide (Klein et al., 2016). Three genomic regions, each with multiple linked loci for phenology (plant height and flowering), have been found to control adaptation of grain type sorghum in temperate zones (Thurber et al., 2013).

Although maize is highly sensitive to low temperature, there is natural variation in freezing and chilling tolerance. The mechanisms responsible for chilling tolerance include modification of photosynthetic apparatus modification, cell wall properties, and developmental processes (Sobkowiak et al., 2016). Differential gene expression in response to freezing identified nine candidate genes with higher expression levels and eight candidate genes with lower expression levels in the tolerant compared to the intolerant lines (Li Z. et al., 2016; di Fenza et al., 2017). Dent and flint maize, which differ in their kernel phenotypes, represent two major temperate gene pools in maize. The flint contributes to early vigor and cold tolerance, while dent increases productivity in hybrids. Unterseer et al. (2016) identified candidate genes under differential selection pressure in these two genepools. Most flint-specific candidate genes were associated with endogenous pathways, whereas dent candidate genes were mainly involved in response to environmental factors such as light and photoperiod.

Low temperature is one of the major constraints limiting rice productivity and cultivation in high-altitude regions. The major rice QTL *COLD1*, which functions as a regulator of G-protein signaling, confers chilling tolerance. The allele SNP2 in *COLD1^jap/ind^* enhances the ability to activate G-protein α GTPase, as *COLD1* interacts with G protein to activate the Ca^2+^ channel for temperature sensing (Ma et al., 2015). A novel gene *CTB4a* controlling cold tolerance at booting stage in rice enhances seed setting and grain yield under cold stress conditions (Zhang et al., 2017). *Oryza glaberrima* has contributed the major QTL *OgTT1*, which confers adaptation to heat stress. *OgTT1* protects cells from heat stress through more efficient elimination of cytotoxic denatured proteins and more effective maintenance of heat-response processes. Overexpression of this gene was associated with markedly enhanced thermotolerance in rice, *Arabidopsis* and *Festuca elata* (Li et al., 2015). Thus, deployment of QTL conferring chilling- and thermo-tolerance is expected to aid development of rice cultivars with enhanced adaptation to these climatic variables.

Soybean was domesticated in temperate regions and is highly sensitive to photoperiod. However, temperate soybean cultivars are not adapted to tropical and sub-tropical climates. The discovery of the long juvenile (LJ) trait in tropical soybean germplasm and its deployment has extended cultivation of temperate soybean to low altitude tropical and sub-tropical climates (Hartwig and Kiihl, 1979; Neumaier and James, 1993). A major locus *J* identified as the ortholog of *Arabidopsis ELF3* confers the LJ trait: *J* promotes flowering, while *j* delays flowering, providing new insight into soybean adaptation to tropical climates (Lu et al., 2017).

The discovery of novel allelic variation and investigation of its genetic and molecular basis has facilitated the successful conversion and adaption of tropical genepools to temperate climates (or temperate genepools to tropical climates) as noted in maize, rice, sorghum, and soybean. This enables exploration of new adaptation niches in agro-ecosystems where farmers are currently growing crops that may become unsuitable due to future climate change.

### Rare Alleles to Benefit Future Genetic Improvement

While common alleles in crops are more likely to be involved in beneficial traits, useful variation may persist as rare alleles that have not undergone strong natural or human selection. The potential agronomic benefits of rare alleles are evident in many traits associated with domestication that are rare in natural populations. For instance, dwarf height, reduced tillering, non-shattering seeds and male sterility alleles will be rare in natural populations. In humans, the search for the ‘missing heritability’ of diseases has led to the understanding that rare alleles (at frequencies as low as <0.1%) can have major phenotypic effects (Fritsche et al., 2016). However, identifying rare alleles in wild or crop populations is challenging because extremely large populations need to be phenotyped and genotyped to detect rare alleles. There is also considerable ascertainment bias against rare alleles because these alleles can be confounded with sequencing errors (Heslot et al., 2013) and routine filtering often excludes alleles with a frequency below 5% to facilitate detection of common variants linked to traits.

Despite the challenges involved in detecting trait-linked rare alleles to use as candidates for crop improvement, substantial progress has been made as the cost of sequencing has decreased and the power of association mapping has risen. A remarkable success was achieved in maize, where the discovery of beneficial rare alleles *LcyE* and *crtRB1* using association mapping (Harjes et al., 2008; Yan et al., 2010) later allowed introgression of the high-provitamin A trait into cultivars consumed in developing countries where vitamin A deficiency in children is an important public health concern (Azmach et al., 2013). In rice, a rare allelic variant of an upstream promoter of *OsglHAT1* was shown to enhance grain weight and yield (Song X.J. et al., 2015), and a rare allele of the grain length QTL, *qGL3*, increases grain length, filling, and weight (Zhang et al., 2012).

### Harnessing Epigenetic Variation for Crop Improvement

Epigenetics is broadly defined as “*the study of mitotically and/or meiotically heritable changes in gene function that cannot be explained by changes in DNA sequence*” (Russo et al., 1996). In the context of nuclear genes, epialleles are epigenetically modified alleles whose function is altered as a result of the particular epigenetic modification(s) (Finnegan, 2002). The range of reversible molecular mechanisms that can generate epialleles includes DNA methylation and a range of possible modifications (methylation, acetylation, phosphorylation, ubiquitination) to histones that can change chromatin states (Pikaard and Scheid, 2014). Additional molecular mechanisms that can generate or alter epialleles involve structural proteins and enzymes involved in chromatin assembly and remodeling (Pikaard and Scheid, 2014).

While transposable elements are key drivers of genetic variation in crop genepools (Bennetzen and Wang, 2014), they are also major drivers of epigenetic variation (Seymour and Becker, 2017; Song and Cao, 2017). In addition, there is a strong interplay between genetic variation (e.g., SNPs) and epiallelic variation (as measured by DNA methylation variation) (Eichten et al., 2013; Meng et al., 2016). Transposon-associated epigenetic variation has been shown to have functional effects in a range of crops, including rice (Zhang X. et al., 2015), melon (*Citrullus lanatus*) (Martin et al., 2009) and oil palm (*Elaeis guineensis*) (Ong-Abdullah et al., 2015).

Unlike mutational changes, epigenetic changes are potentially reversible. For instance, research on paramutation (since Brink’s pioneering studies of the *R* locus in maize) showed that at some loci, one allele can induce a heritable epigenetic change in the other allele (Brink, 1958; Hollick, 2016). Similar reversible epigenetic states are evident for loci regulated by genomic imprinting (Garnier et al., 2008), nucleolar dominance (Tucker et al., 2010) and gene silencing (Pikaard and Scheid, 2014). Indeed, epigenetic studies in plants (e.g., for the *Lcyc* and *FWA* loci) demonstrated that epialleles associated with biological functions can be heritable over multiple generations (Cubas et al., 1999; Soppe et al., 2000). Fundamental research using genetically identical yet epigenetically diverse recombinant inbred lines (epiRILs) has shown that epigenetic variation is associated with phenotypic variation and that such epiRILs can remain stable over generations (Johannes et al., 2009), at least in inbreeding plant species. Epigenetic quantitative trait loci (epiQTL) have been identified associated with traits such as root length and flowering time (Cortijo et al., 2014; Kooke et al., 2015). It has been also demonstrated that RNA interference (RNAi) pathways are important to maintain DNA methylation pattern fidelity over generations (Teixeira et al., 2009).

While it is clear that epialleles contribute to functional effects in plants, that can be trans-generationally inherited and be reversible (e.g., in response to abiotic or biotic environmental stimuli) (Becker et al., 2011; Schmitz et al., 2011), it is also emerging that epigenetic variation (epialleles) and mechanisms can potentially make contributions to functional traits in crop genepools (Eichten et al., 2011; Li Q. et al., 2014). Plant epigenome diversity research revealed that while different geographic origins display different genome-wide DNA methylation levels and epiallelic gene expression (Kawakatsu et al., 2016), there is no detectable signal of DNA methylome adaptation to the environment (Hagmann et al., 2015). In addition, the contribution of the DNA methylome to gene expression regulation has been demonstrated to be much less than the contribution from SNPs (Meng et al., 2016). While over a 1000 expression traits displayed significant SNP associations, less than 60 of these displayed an association with DNA methylation polymorphisms (Meng et al., 2016). Such findings have implications for crop improvement, particularly if epiallelic variation is contributing to specific adaptation of crops or their wild relatives to agro-environments.

Heterosis refers to the superior performance of F_1_ progeny compared to their parents and is extensively harnessed for crop improvement (Duvick, 2001). While heterosis in plants may be due to genetic dominance (complementation), overdominance and pseudo-overdominance effects (Birchler et al., 2010; McKeown et al., 2013; Schnable and Springer, 2013), there is emerging evidence that epigenetic variation and mechanisms may contribute to heterosis effects in plants (Shivaprasad et al., 2012; Groszmann et al., 2013; Offermann and Peterhansel, 2014). For instance, “heterosis without hybridization” has been demonstrated in plants using epiRILs and triploid lines that are genetically identical but epigenetically different (Duszynska et al., 2013; Dapp et al., 2015; Fort et al., 2016).

Metastable epialleles are alleles that display variable expressivity despite being in an identical genetic background. Research using metastable epialleles of the red color *r1* locus in maize did not, however, support a metastable epigenetic contribution to heterosis or inbreeding depression (Auger et al., 2004). While non-additive DNA methylation effects have been observed in F_1_ hybrids that display heterosis (Greaves et al., 2014), the functional significance of such DNA methylation changes is unclear, as genetic ablation of such RNA-mediated DNA methylation interactions did not affect heterosis biomass, while the chromatin remodeller *DDM1* has been identified as a modifier of heterosis (Groszmann et al., 2011; Shen et al., 2012; Kawanabe et al., 2016; Zhang et al., 2016a,b).

Apomixis refers to asexual reproduction via plant seeds. Despite being a naturally occurring phenomenon, the fixation of heterosis via apomixis to generate true-breeding lines in crop improvement programs has been extensively proposed but has not been realized to date (Spillane et al., 2004). Indeed, while it was expected that apomixis could fix F_1_ heterosis effects that have a genetic basis, this had not been demonstrated until recently where it has been shown that apomixis could fix 90% of traits generated in *Pilosella* F_1_ hybrids over two successive generations (Sailer et al., 2016).

Research in tomato (*Solanum lycopersicum*) and emerging studies on other fleshy fruit crops are revealing a role for epigenetic control of fruit ripening (Gallusci et al., 2016), e.g., DEMETER-like DNA demethylases and *CHROMOMETHYLASE3 (SICMT3)* genes regulate fruit-ripening associated transcription factors (e.g., *RIN*) and epi-alleles (*Cnr*) in tomato (Manning et al., 2006; Zhong et al., 2013; Chen et al., 2015; Liu R. et al., 2015). Epigenetic variation in crop genepools at loci that are sensitive to environmental signals (e.g., temperature, vernalization, photoperiod, flowering time, and ripening) has potential for more effective harnessing through breeding (King, 2015), involving selection of functional epiallelic variants, and potentially future epi-genome editing (Klann et al., 2017).

Epigenetic variation that contributes to adaptive phenotypic variation may be particularly important in fluctuating environments, and could play an important phenotypic plasticity role as a buffer to environmental stimuli, and both abiotic and biotic stresses. For instance, tight epigenetic regulation of the antagonistic NLR receptors *PigmR* and *PigMS* is necessary to confer rice blast resistance with minimal yield penalty (Deng et al., 2017). The identification of such epialleles and epigenetic regulatory systems conferring functional impacts on agronomic traits can feed into a range of different approaches emerging for epigenetic breeding of crop plants, including use of mutant lines (Yang et al., 2015), recurrent epi-selection (Hauben et al., 2009; Greaves et al., 2015), hybrid mimics (Wang L. et al., 2015), epigenomic selection (Jonas and de Koning, 2013; Oakey et al., 2016) and epigenome editing (Park et al., 2016).

Many studies on the relationship between epigenetic variation and epialleles and phenotypic variation (including expression variation) have been conducted under controlled conditions. As a result, little is known of the extent of epigenotype × genotype × environment (epiG × G × E) interactions of crops under field conditions. For crop improvement, harnessing both multilocus epiallele interactions (with associated epistasis effects) and single locus “major effect” epialleles offer opportunities for developing novel approaches for increased epigenetic gain in crop breeding programs.

## Harnessing Functional Diversity With New Trait Improvement Technologies

### Genomic-Estimated Breeding Values to Predict the Utility of Germplasm Accessions

While there are 100s or 1000s of germplasm accessions conserved in *ex situ* genebanks globally, the lack of phenotyping and genotyping data limits their use. Advances in genomics, phenomics and bioinformatics are increasing the availability and quality of data to better leverage this germplasm for breeding (McCouch, 2013). Association genetics along with genomic prediction further allows expansion of use of genetic variation, with the aim of increasing yield-related genetic gains in cereals (Spindel et al., 2016). Ayling (2016) provides an overview of promising methods for increasing the knowledge on (and utility of) genebank accessions using next generation sequencing (NGS). Moreover, emerging cross-disciplinary “genoplasmics” has been proposed as new term to refer to genomics-assisted plant germplasm research (Jia et al., 2017). Such a research methodology involves defining core collections or core subsets (that capture maximum evolutionary history in a limited number of accessions) are promoted for genetic enhancement or gene discovery (van Hintum et al., 2000). For example, investigation of spring bread wheat diversity (in a genebank from mega environments) by high quality genotyping-by-sequencing (GBS) loci and gene-based markers permitted selection of novel variation for further use in breeding crops with traits such as adaptation to drought or heat stress (Sehgal et al., 2015b).

Longin and Reif (2014) proposed a stepwise strategy for better use of wheat genetic resources that are available in genebanks. They propose using representative core subsets of accessions that are defined after genotyping and assessing their genetic relationships, including consideration of whether different accessions harbor major adaptation genes to stressful environments. Specific accessions are selected according to phenotyping and genome-wide data; i.e., genotypic and phenotypic data are used to estimate effects for all genomic regions and to develop models for predicting genomic estimated breeding values (GEBV) of genebank accessions that may be candidate parents of elite wheat breeding lines. This approach targets the entire genome rather than focusing on major genes with large effects related to traits of interest. Genomic prediction models including the genotype × environment interaction have already been shown to be promising for introgressing highly heritable traits from exotic wheat landrace germplasm stored in genebanks into elite breeding lines (Crossa et al., 2016). These results validate the direct use in crop breeding of the substantial landrace genetic diversity that is conserved in genebanks. This introgression breeding approach also requires GEBV to predict the value of the resulting offspring. In the last step of the proposed strategy, genotypic and phenotypic data along with passport and pedigree information are shared through a database platform to facilitate breeding.

As noted by Brown (2016), the major interest of using GEBV for predicting traits lies on replacing expensive phenotyping with inexpensive genotyping. In this regard, Yu et al. (2016) provided a proof-of-concept study that integrated genomic prediction into the evaluation of germplasm with a broad genetic base. They first characterized a sorghum core subset (962 accessions) with GBS. Next, 299 accessions representing the overall diversity of the core subset were selected as a training set for biomass yield and other related phenotypic traits such as plant height, stalk number and root lodging, amongst others. Cross-validation demonstrated a high prediction accuracy for stalk number and biomass yield. Similarly, Gorjanc et al. (2016) used GEBV to harness multigenic variation from maize landraces. Their results suggest that genetic enhancement using high levels of genetic diversity can begin directly with landraces. They also indicated that early introgression into elite germplasm seems to be feasible for loci with large effects, but not for landrace haplotypes harboring multi-genic variation because further improvement will favor the elite haplotypes and limit the distinctness of resulting germplasm. Similarly, Burstin et al. (2015) were able to predict flowering, seeds per plant, and seed weight using diverse pea accessions after characterizing the accessions with DNA markers. Genomic prediction, as shown by Jarquin et al. (2016), depends on having both the target population and environment in the training set, and on including data from diverse geographical locations and genetic clusters. Their research highlights the value of historical germplasm data to develop predictive models that assist in selecting genebank accessions for introgressing useful genetic variation into breeding populations and programs.

### CRISPR/Cas9 to Release Novel Variation

Genome editing is a rapidly emerging targeted mutagenesis approach that offers unique opportunities to elucidate gene functions and introduce novel beneficial alleles into crop germplasm (Voytas, 2013; Scheben and Edwards, 2017). Although engineered nucleases such as transcription activator-like effector nucleases (TALENs) and zinc finger nucleases (ZFNs) can be used for genome editing, the dominant tool at present is the type II clustered regularly interspaced short palindromic repeat (CRISPR)/CRISPR-associated protein (Cas) system using the Cas9 nuclease (Jinek et al., 2012). The CRISPR/Cas system targets specific genomic regions via a guide RNA which hybridizes to a G(N)_19-22_NGG target DNA sequence downstream of an NGG protospacer adjacent motif (Gasiunas et al., 2012; Jinek et al., 2012). The guide RNA forms a complex with the Cas9 nuclease which then cleaves double stranded DNA at the target site. The error-prone non-homologous end joining (NHEJ) DNA repair pathway repairs the double-strand break, typically introducing a deletion. By delivering a DNA repair template together with the CRISPR/Cas system, in principle precise insertions or deletions can be achieved via the error-free homology directed repair (HDR) pathway.

CRISPR/Cas has now been demonstrated to improve agronomic traits in numerous crops. For example, genome edited lines of the rice genes *gn1a*, *dep1*, *gs3*, *dep1* and *gs3* showed enhanced grain number, dense erect panicle, larger grain size, semi-dwarf stature and long-awned grains, respectively (Li M. et al., 2016). Disrupting pest-susceptibility genes increased resistance to fungal blast in rice (Wang F. et al., 2016), resistance to powdery mildew in bread wheat (Wang Y. et al., 2014) and broad-spectrum disease resistance in tomato (de Toledo Thomazella et al., unpublished). The CRISPR/Cas system has also unraveled several biallelic mutations of *Glyma06g14180* and *Glmya08g02290* with varying gene expression during hairy root development in soybean (Sun et al., 2015). While these studies have relied on disruption of target genes via error-prone NHEJ to change plant phenotypes, progress has also been made in the more difficult to achieve precise gene targeting using HDR. In a recent study in maize, a promoter of the drought-tolerance associated *ARGOS8* gene was swapped using CRISPR/Cas and HDR with the U3 maize promoter to increase expression of the *ARGOS8* gene (Shi et al., 2016). Field trials showed that genome edited maize plants had significantly higher yield under drought stress and no yield loss under normal conditions.

The major current limitations in the application of CRISPR/Cas genome editing for crop improvement relate to the inefficient precise gene targeting via HDR (Steinert et al., 2016). However, targeted base editing has now been demonstrated in rice, wheat, maize and tomato using Cas9-cytidine deaminase fusions (Zong et al., 2017; Shimatani et al., 2017). In addition, given that most cereal and legume crops are auto- or allo-polyploids, genome editing now offers the exciting opportunity for targeted mutagenesis in polyploid plant genomes (Ryder et al., 2017), which has not been possible with conventional mutagenesis techniques (Kumar et al., 2017). While there has been some focus on potential lack of target site specificity (Fu et al., 2013; Jiang W.Z. et al., 2013; Pattanayak et al., 2013) from early generation CRISPR/Cas systems, improved systems are under development to minimize any off-target edits. For instance, innovative techniques such as paired Cas9 nickases (Ran et al., 2013) and highly specific Cas9 variants (Kleinstiver et al., 2016) are increasing the DNA target specificity of genome editing. In any event, weighing up the benefits of gene targeting against any (hypothetical) costs associated with off-target editing in crop genomes, very low levels of off-target editing in crop genomes is unlikely to be of any major concern, given that (chemical and radiation) mutagenized lines have been used for decades for crop improvement. Genome editing has major potential not only for crop improvement but also for rapid domestication of novel crops from wild species or minor crops by simultaneously editing genes related to domestication such as grain size, shattering, plant stature, and flowering time. As wild plants may harbor greater diversity in climate-related traits such as stress tolerance and pest resistance, CRISPR/Cas-assisted breeding approach may play an important role in increasing global food production in a changing climate.

## Storage and Integration of Genetic and Phenotypic Information

Although vast amounts of sequence and expression data are hosted by the European Molecular Biology Laboratory (EMBL) (Kanz et al., 2005), GenBank (Benson et al., 2012), and the DNA Data Bank of Japan (DDBJ) (Mashima et al., 2016), crop improvement relies on the integration of such data with more widely dispersed data on sequence variation and phenotypes. Storing and managing the increasing amounts of public and private genotypic and phenotypic data on crops, however, is challenging (Batley and Edwards, 2009; Lee et al., 2012). In the past decade or so, crop-specific databases and bioinformatics services have been developed for many crops (**Table 7**). These public databases provide access to genomes and the corresponding annotation data, together with data on phenotypes and genotypes. Tools such as ngs.plot (Shen et al., 2014) and QTLNetMiner^2^ enable integrated analysis of genotype and phenotype information contained in databases. Mining of genomic databases in this way or using more advanced machine learning approaches can facilitate discovery of genes related to specific target functions. A range of databases for the management of crop germplasm have also been developed by the European Cooperative Programme for Plant Genetic Resources Networks (ECPGR) and the United States. National Plant Germplasm System (NPGS). Linking germplasm information to broader genetic and phenotypic resources would allow easier accessibility of germplasm for experimentation.

**Table 7:** Databases integrating crop data including genomes, genotypes and phenotypes.

Database	Crops	Web link	Reference
Gramene	Grasses	http://www.gramene.org	Tello-Ruiz et al., 2016
International Rice Informatics Consortium	Rice (*Oryza sativa*)	http://iric.irri.org	n/a
Maize Genetics and Genomics Database	Maize (*Zea mays*)	http://www.maizegdb.org	Lawrence et al., 2004
SoyKB	Soybean (*Glycine max*)	http://soykb.org	Joshi et al., 2014
T3 Triticaceae toolbox	Wheat (*Triticum aestivum*), barley (*Hordeum vulgare*) and oat (*Avena sativa*)	https://triticeaetoolbox.org/	Blake et al., 2016
Wheat Information System	Wheat (*Triticum aestivum*)	http://wheatis.org	n/a

The large scale of genotypic and phenotypic data requires a powerful computational platform for data management and parallel processing. The open-source Apache Hadoop framework suits the demands of large-scale processing of genomic data (Niemenmaa et al., 2012; Nordberg et al., 2013; O’Driscoll et al., 2013). Cloud computing services such as those provided by Amazon (Madduri et al., 2014), or institutional dedicated computing clusters, allow researchers cost-effective access to the computational power required for integrated analysis of large biological datasets. While the infrastructure for developing databases to host and help analyze big biological data is available, long-term funding of database projects is rare, despite being essential infrastructure to maintain and curate the continuously growing databases. Moreover, the usefulness of existing databases is often severely limited by a lack of phenotype data because the generation of genotypic data has fast outstripped that of other data types. Without phenotype data, it is not possible to carry out association genetics or identifying candidate genes linked to agronomic traits (Cobb et al., 2013). Advances in phenomics such as remote sensing, robotics and automated environmental data collection may help overcome the bottleneck in phenotyping data (Furbank and Tester, 2011; Araus and Cairns, 2014).

Another step required to facilitate the integration of large-scale crop data is the use of shared vocabularies for genetic and phenotypic information. The gene ontology (GO) project has made major strides in the use of universal vocabularies for the annotation of genes, gene products and sequences. Nevertheless, the vocabularies used for describing sequence variation and phenotypic traits remain inconsistent and hamper data integration. Researchers must collaborate closely in developing consistent vocabularies to accelerate the development of broadly informative crop databases that are useful for plant breeders.

Finally, an important step for crop improvement would be an increase in data sharing between public and private sector institutions (Spindel and McCouch, 2016). Political disagreements on access to crop genetic resources and the distribution of benefits have led to protectionist attitudes regarding plant genetic resources, indicating the need for an acceptable data sharing framework. The 2001 International Treaty on Plant Genetic Resources for Food and Agriculture (IT PGRFA) provides a multilateral framework (agreed between the world’s governments) for exchange of plant genetic resources (using a common material transfer agreement (MTA) for each accession) between countries (and their institutions). The IT PGRFA covers access to the vast majority of the world major crop and forage species, which are listed in Annex 1 of the Treaty. Future challenges will undoubtedly emerge regarding access and benefit sharing relating to genomic or phenomic data derived from crop genetic resources, which fall under the auspices of the International Treaty on Plant Genetic Resources.

## Current Knowledge and Future Challenges in Functional Diversity

Advances in genomic technologies have led to an unprecedented availability of crop sequences and sequence variation data. As crop genomes are re-sequenced to better represent the genetic diversity in the gene pool, pangenomes capturing core and variable genes in crop species are becoming available, e.g., in maize (Hirsch et al., 2014), rice (Schatz et al., 2014), wheat (Montenegro et al., 2017), soybean (Li Y.H. et al., 2014), *Brassica rapa* (Lin et al., 2014), and *Brassica oleracea* (Golicz et al., 2016). Despite this wealth of genomic data, gene functions and networks remain very poorly characterized, even in crops such as rice (Rhee and Mutwil, 2014). The genetic mechanisms controlling important agronomic traits are only slowly being elucidated, revealing complex interaction networks and considerable diversity between crops, as in the case of the universal florigen flowering pathway (Turck et al., 2008). The genes underlying other complex traits such as abiotic stress adaptation are still only partly known and often confounded by gene by environment interactions (Fleury et al., 2010). Association mapping of these complex traits, combined with reverse genetic screening to elucidate trait-gene associations, will be crucial to uncover both common and rare alleles of yield-related traits.

While trait-gene association is currently hampered by a lack of extensive phenotype data from well characterized environments, recent advances in high-throughput phenotyping platforms that can be used in the field may soon help overcome this challenge (Furbank and Tester, 2011; Araus and Cairns, 2014). By utilizing the diverse germplasm resources available from crops and their wild relatives to uncover genes that can be introgressed into elite breeding germplasm, crops can continue to be improved for potential yield and yield stability. An important step to accelerate such breeding efforts worldwide is the integration of this information in openly accessible databases, which is currently lagging behind the rapid generation of data. As climate change and a growing population put increasing pressure on plant breeders in the public and private sectors to produce high-yielding, climate resilient cultivars, a consensus needs to also be reached on the merits of genome editing to produce novel and useful diversity in crop germplasm, to rapidly improve agronomic traits associated with known genes. It is increasingly clear that crop improvement must draw on diverse germplasm pools and leverage advances in biotechnology to ensure future global food security.

## Conclusion

**FIGURE 1:**
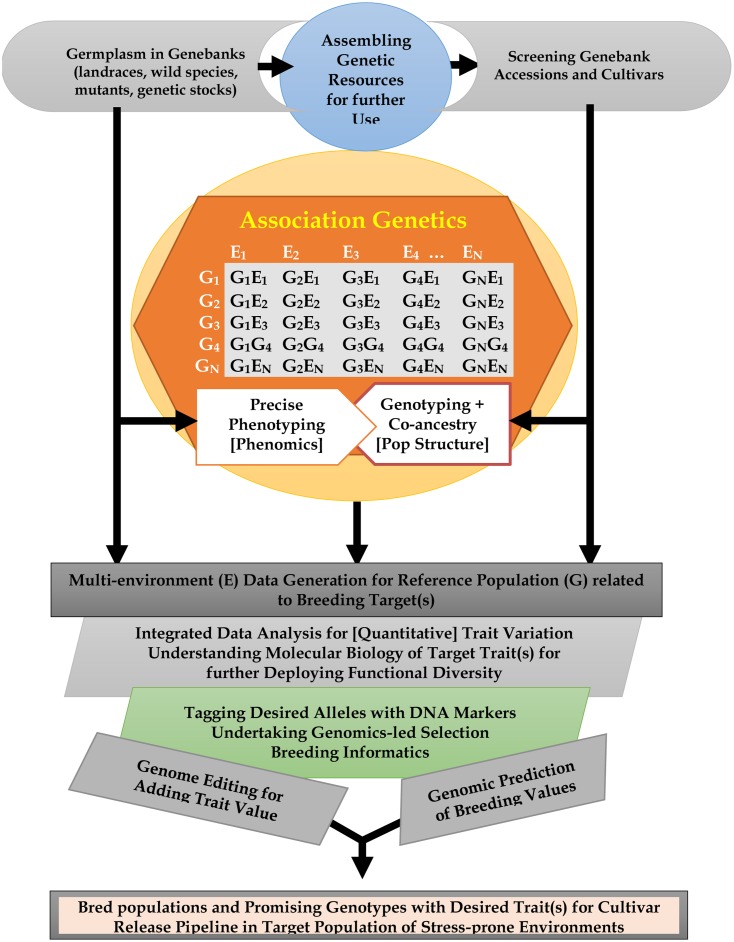
Assessing and exploiting functional diversity in germplasm pools in the omics era for plant breeding under a changing climate.

Plant genetic resources provide raw materials for mining allelic variations associated with target traits (**Figure 1**). Crop improvement continues to rely on combining diversity in crop populations and their wild relatives via genetic recombination. Sequencing technology advances and bioinformatic tools used for assessing diversity in germplasm panels have identified millions of polymorphic SNPs in cereals and legumes, as noted by the examples included in this article. Unlocking functional diversity for key agronomic traits such as crop phenology, plant architecture, yield and stress tolerance is facilitating greater use of germplasm in crop breeding. Major QTL and candidate SNPs associated with such agronomi traits, identified through genome wide association research (and ideally confirmed by functional studies), have been deployed in crop breeding to enhance adaptation and productivity of staple food crops. The discovery and deployment of alleles associated with variation in response to photoperiod and flowering has allowed the cultivation of tropical crops such as maize, rice and sorghum in temperate climates (*CTB4a*, *Ghd7*, *PRR37*, *RFT1*, *Sb06g012260*) or temperate crops such as soybean in tropical regions (*J* locus).

Flowering time is a major determinant of crop yield, and selection of variants in florigen pathway and their deployment in crop breeding has increased yield in several crops. In particular. fine-tuning of the florigen pathway has allowed yield increases through better control of flowering and growth in crops. Grain legumes have indeterminate flowering, leading to low productivity compared to cereals. The discovery of candidate genes, *Phvul.001G189200* in common bean and *CcTFL1* in pigeonpea associated with determinate flowering may allow manipulation of determinacy trait in these and other legumes.

Functional allelic diversity has been successfully harnessed through breeding to enhance abiotic stress (drought, salinity, low soil P, and submergence tolerance) adaptation and productivity by manipulating panicle architecture in rice. Likewise, QTL hotspots associated with drought adaptation in chickpea have been introgressed in several leading cultivars in Asia and Africa. *Glycine soja* is an interesting source of variation for abiotic stress adaptation in soybean. Candidate SNPs associated with drought adaptation are known in common bean. Changes in functional diversity due to global warming were noted for flowering in wild barley and emmer wheat from Israel or among pearl millet landraces from Africa, thus providing valuable resource for enhancing crop adaptation to variable climates leading to shortening of growing season. Genomics have unraveled SNPs associated with precipitation and length of growing season in barley or with precipitation and high temperature in maize and sorghum, with many located in genes known for abiotic stress adaptation, thus providing valuable resource to accelerate breeding for drought-prone environments.

Maize has comprehensive haplotype maps that has enabled researchers to identify selective sweeps and chromosome regions harboring loci related to domestication and geographic adaptation. Identification of distinct haplotypes in subtropical maize germplasm provided opportunity to exploit heterotic potential among them. Likewise, distinct haplotypes discovered amongst Indian wild rice accessions associated with high salinity tolerance from distant geographic regions may be useful for broadening the available cultigen pool to enhance rice productivity in salt-prone areas.

Landraces and wild relatives are proven genetic resource to identify genetic variants associated with environmental adaptation, particularly temperature and precipitation. In addition, such genetic resources are also the source of discovering rare alleles; however, identifying such alleles is challenging because of their low presence in populations, necessitating phenotyping and genotyping of extremely large populations. Evidence suggests that epigenetic variation (i.e., epialleles) can also be successfully exploited to enhance abiotic stress adaptation and productivity in crops. Genome editing in maize, rice and soybean or estimating genomic-estimated breeding values of genebank accessions in maize, pea, sorghum, and wheat provide means to access and generate additional variability for agronomic and stress tolerance traits.

Phenomics and genomics are enabling generation of vast data sets in crop breeding. However, archival and easy retrieval of these data set is a challenge. Crop-specific databases along with bioinformatics services provide access to genomes and the corresponding annotation data, together with data on phenotypes and genotypes for many crops. An integrated analysis of genotype and phenotype information contained in databases facilitates the discovery of genes related to specific target functions. Such insights on crop biodiversity and trait inheritance along with mapping of genetic variation controlling key traits, and using them for developing breeding germplasm will accelerate crop improvement, increase genetic gains and allowing improved crop yields and yield stability under a changing climate and in stress-prone environments.

## Author Contributions

All authors participated in outlining the manuscript contents, searching the literature, writing and editing the text.

## Conflict of Interest Statement

The authors declare that the research was conducted in the absence of any commercial or financial relationships that could be construed as a potential conflict of interest.

## Footnotes

1 http://www.ars-grin.gov

2 http://ondex.rothamsted.ac.uk/QTLNetMiner/
